# Macrofaunal Responses to Edges Are Independent of Habitat-Heterogeneity in Experimental Landscapes

**DOI:** 10.1371/journal.pone.0061349

**Published:** 2013-04-08

**Authors:** Miguel G. Matias, Ross A. Coleman, Dieter F. Hochuli, Antony J. Underwood

**Affiliations:** 1 Centre for Research on Ecological Impacts of Coastal Cities, Marine Ecology Laboratories A11, School of Biological Sciences, The University of Sydney, Sydney, Australia; 2 School of Biological Sciences, The University of Sydney, Sydney, Australia; University of Southampton, United Kingdom

## Abstract

Despite edges being common features of many natural habitats, there is little general understanding of the ways assemblages respond to them. Every edge between two contrasting habitats has characteristics governed by the composition of adjoining habitats and/or by the nature of any transitions between them. To develop better explanatory theory, we examined the extent to which edges act independently of the composition of the surrounding landscape and to which transitions between different types of habitats affect assemblages. Using experimental landscapes, we measured the responses of assemblages of marine molluscs colonising different experimental landscapes constructed with different compositions (i.e. different types of habitats within the landscape) and different types of transitions between habitats (i.e. sharp vs gradual). Edge effects (i.e. proximity to the edge of the landscape) were independent of the internal composition of experimental landscape; fewer species were found near the edges of landscapes. These reductions may be explained by differences in differential larval settlement between edges and interiors of experimental landscapes. We also found that the sharpness of transitions influenced the magnitude of interactions in the different types of habitats in experimental landscapes, most probably due to the increased number of species in areas of transition between two habitats. Our experiments allowed the effects of composition and transitions between habitats to be disentangled from those of proximity to edges of landscapes. Understanding and making predictions about the responses by species to edges depends on understanding not only the nature of transitions across boundaries, but also the landscape in which the edges are embedded.

## Introduction

Ideas from landscape ecology have advanced our conceptual understanding of how boundaries between and within habitats may mediate biotic interactions with surrounding habitats [see reviews in 1,2]. Although it is assumed that edge-effects are generally apparent among different systems, there is a lack of consistency in studies on edges [see review by 3]. In fact, it is quite common to find reports of unidirectional edge-effects regardless of the nature of the adjoining habitat, which is often assumed to be an unsuitable matrix [reviewed by 4]. The responses of biota to edges are, however, often dependent on the composition of the landscape in which the edge is embedded [e.g. 5,6]. There can be no generality in understanding of biotic responses to edges until the effects of edges themselves are disentangled from influences due to composition of a landscape (i.e. varieties or amounts of different types of habitats in the landscape).

Unpredictability in biotic responses to edges might arise because many boundary-related concepts (e.g. edge, border, boundary, ecotones and corridors) are often used interchangeably [see review by 7], which reduces the chance of detecting generality across studies and systems. Edges are usually defined as boundaries between distinct types of habitat [8, or “systems”, 9], or as areas of a habitat near a perimeter adjoining an unsuitable matrix [10]. In these cases, the nature of the surrounding habitats (i.e. matrix) may influence the response of organisms to edges [8]. Edges between different types of habitats can also be influenced by their “sharpness” [9]. Sharp (or hard) edges are abrupt changes in structural complexity between two types of habitat, whereas gradual (or soft) edges reflect smoother transitions between different habitats [11]. Hereafter, we will use “edges” to refer to areas near the perimeters of a habitat in contact with an unsuitable matrix. “Transitions” are boundaries between different habitats (some of which might be unsuitable for some species) within a landscape.

Although most studies and theoretical syntheses on effects of boundaries have been on terrestrial systems [7], [9], [10], [11], there has been increasing interest in extending our understanding of marine landscapes such as seagrass beds [e.g. 12,13,14,15], mangroves [e.g. 16,17] and macro-algal beds [e.g. 18,19]. Boundary-related effects in marine habitats are often context-dependent [see review in 13]. For instance, it has been shown that the effect of distance from an edge of habitat is dependent on the size of habitats – large areas might show edge-effects, whilst small areas do not [e.g. 20,21,22,23]. Furthermore, the direction of such effects might also be species-specific: fish assemblages in areas of seagrass can vary according to the distance to an edge [e.g. 22,24], while mobile epifauna do not vary across such areas [25]. Interestingly, similar habitats may reveal edge-effects in different directions: some areas of seagrass have greater faunal abundances in edges than in their interiors [e.g. 26,27]. Other studies, however, have found fauna to be less dense at the edge than in inner regions of seagrass beds [e.g. 24,28]. Such disparity of results emphasises that the detection and interpretation of edge-related effects are closely linked with the scale and structure of landscapes and with intrinsic properties (e.g. recruitment, mode of dispersal, etc.) of the associated fauna.

Experimental landscapes have been extensively used to investigate patterns of diversity in heterogeneous habitats such as bushes [29], mosses [30], freshwater ponds [31], microphytobenthos [32], [33] and artificial seagrasses [23]. These experiments have allowed tests of hypotheses that cannot be formally tested by observational studies. Here, we investigate a series of boundary-related effects using a system of experimental landscapes that are colonized by assemblages of marine molluscs associated with structurally complex coralline turfs. This system has been extensively used to investigate the mechanisms underlying patterns of distribution of species in fragmented habitats, including colonization [34], species-area relationships [35] and responses to different compositions of habitats [36]. At small scales, many molluscs show great variability in abundances [37], which has been attributed to recruitment and/or mortality [38] or short-term dynamic patterns of immigration and emigration among habitats [39] and species-specific preference for particular types of habitats [40].

In this study, we examined the concepts that the effects on assemblages of distance to an edge of habitats are independent of the composition of the landscape (i.e. types of habitats within the landscape) and whether they are influenced by the nature of transitions (i.e. sharp vs gradual) between habitats inside the landscape. In particular, we tested the predictions that (1) the diversity of benthic assemblages near an edge differs from diversity in interiors of experimental landscapes, regardless of the composition of experimental landscapes (hypothesis 1); (2) assemblages differ depending on the type of transitions between adjoining patches; and (3) assemblages colonizing whole experimental landscapes are affected by the composition and types of edges within the landscape (hypothesis 3). These predictions were tested using assemblages of molluscs colonizing artificial landscapes, with different composition and types of transition between different habitats in the landscape. Artificial habitats made of synthetic turfs are valid and useful tools for investigating relationships between habitats and their associated molluscan assemblages, because they are colonised by a diverse assemblage of molluscs similar to that found in natural habitats. They also reproduce particular features of natural habitats (e.g. they have dense or sparse fronds), but have more controllable variability among experimental units than is shown by natural macro-algae [e.g. 34,36,41].

## Methods

### Study sites

This study was done in the Cape Banks Scientific Marine Research Area (NSW, Australia, 34° 00′S 150°15′E; NSW Fisheries research permit F96/146-6.0) between November and December 2008. We selected two sites with similar orientation and exposure to waves on gently sloping, low-shore rock platforms, or large boulders, at 0.3 to 0.6 m above mean low water. These sites were occupied mostly by algal turfs dominated by *Corallina officinalis* Linnaeus 1758, which comprised >85% of the areas used. Such beds of turfs can extend over several hundreds of metres or can be highly fragmented patches <0.25 m^2^ [42], [43]. Patches of macro-algae *Hormosira banksii* (Turner) Decaisne 1842 and the colonial ascidian *Pyura stolonifera* Heller 1878 were also present, but in smaller abundances. These species provide biogenic habitats that support diverse assemblages of benthic macro-invertebrates [e.g. 44,45,46], providing a diverse pool of species of potential colonists to these artificial habitats.

### Experimental landscapes

We used three types of synthetic turfs (Grassman Pty Ltd., NSW, Australia; hereafter turfs) as mimics of habitats with different density (D) and overall surface area (SA) of fronds: dense habitats (D = 16.2 fronds.cm^−2^; SA = 25.9 cm^2^), intermediate habitats (D = 12.2 fronds.cm^−2^; SA = 19.4 cm^2^) and sparse habitats (8.1 fronds.cm^−2^; SA = 12.9 cm^2^). All types of turf were 4 cm long. They allowed us to test the effects of changing structural complexity of habitats by manipulating the density of fronds. Previous results have demonstrated that different habitats made of different densities of fronds are consistently colonized by different benthic assemblages [35,e.g. 36].

Investigating the consequences of changes in habitats requires a clear understanding of the scale at which target species respond to such changes [e.g. 47,48]. We used squares of synthetic turf of 5×5 cm as sample size to be consistent with previous experiments that had shown significant differences between adjoining samples of different types of turfs [e.g. 36,49]. Because testing our hypotheses required experimental units that were big enough to be able to manipulate boundaries between habitats and between landscapes and the inhospitable surrounding matrix we used experimental landscapes that were 1800 cm^2^, 6 times larger than the biggest artificial habitats used before [e.g. 35]. This size ensured that the distance between edges and the middles of experimental habitats was four times the length of our previous experimental habitats (i.e. 20 cm).

**Figure 1:**
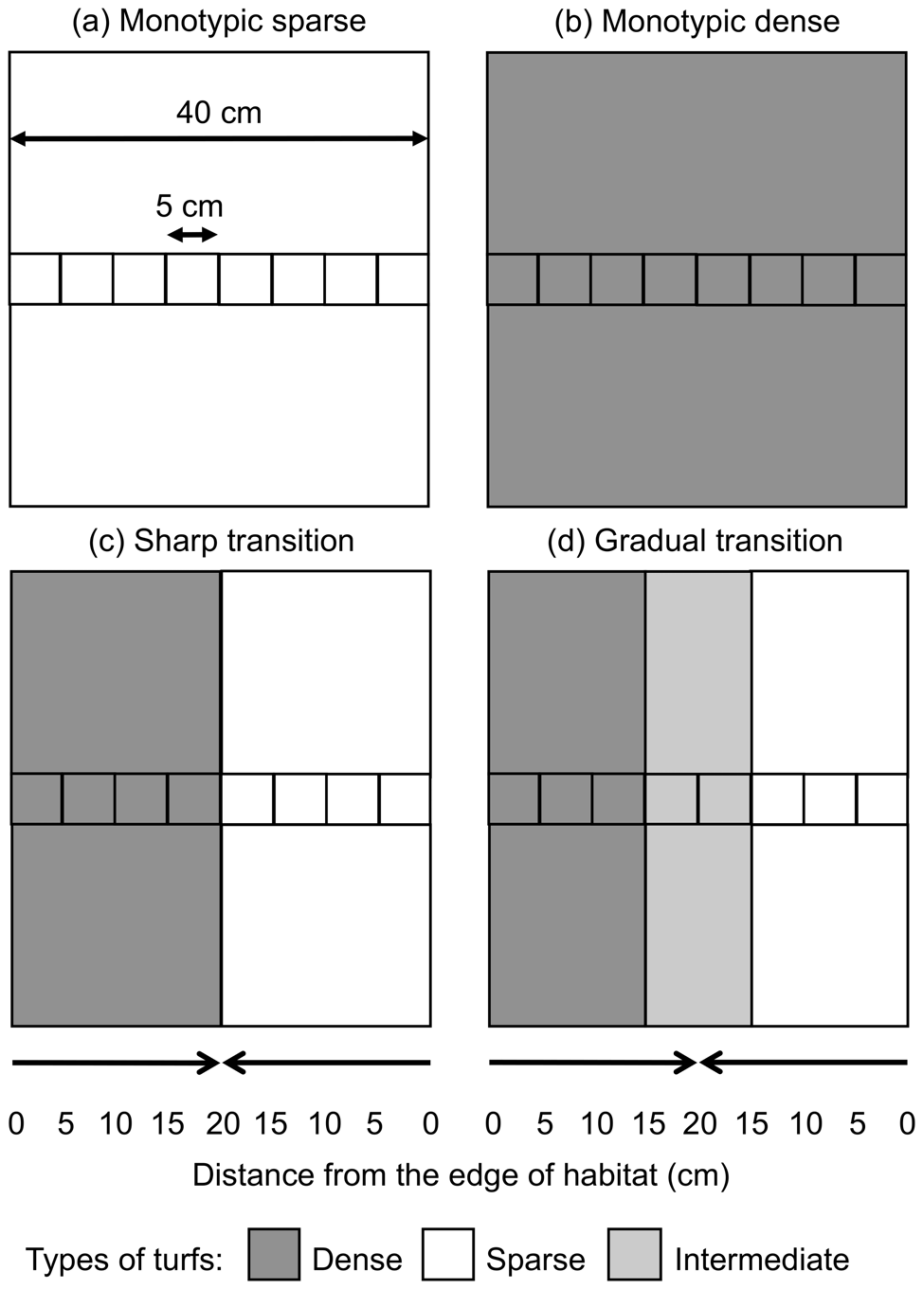
Experimental landscapes of different habitats and with different transitions. (a) Monotypic sparse and (b) monotypic dense; (c) sharp transition between dense and sparse and (d) gradual transition between dense and sparse. The smaller squares in that run across the experimental landscapes represent eight 5×5 cm pieces of artificial turf that were sampled. The final shape of the experimental landscapes was rectangular (40×45 cm; see Fig. 1) to ensure that all eight samples had the same distance to the remaining edges of the landscape (i.e. 20 cm). The area of experimental landscapes was 1800 cm^2^.

We built our experimental landscapes so that it would be possible to sample particular types of habitats across a “transect” than runs through the experimental landscape from one edge to the other. For this purpose, the central part of the experimental landscape (i.e. the “transect”; see Fig. 1) consisted of a row of eight individual pieces of turfs that could easily be dislodged to allow sampling or organisms in different parts of the experimental landscape. These individual pieces of turfs were glued to individual pieces of rubber previously attached to the plastic mesh using a cable-tie, which allows a quick and easy release by removing the cable-tie from each turf separately, without compromising the integrity of each sample [e.g. 36,49]. The remainder of each landscape was completed with turfs of the relevant type that were similarly attached to the wire mesh (see Fig. 1a, b, c and d).

### Experimental design

Four types of experimental landscapes with different compositions were constructed: monotypic sparse (Sparse); monotypic dense (Dense); dense and sparse turfs with a sharp transition (Sharp); dense and sparse with a gradual transition (Gradual; see Fig. 1). Note that the inclusion of intermediate turfs in Gradual habitats does not change the overall surface area of the entire experimental landscapes was similar to that in Sharp habitats. Due to logistic constraints due to the number of experimental units already necessary in the experiment, it was not possible to include the additional treatments solely made of intermediate turfs. This in no way compromised the test our primary hypothesis. These experimental landscapes were constructed so that it was possible to collect samples from four different distances from an edge (0–5, 5–10, 10–15 and 15–20 cm) on each side of the experimental habitats. All experimental landscapes were attached to clear areas of rock with stainless steel screws and rubber washers. This procedure minimized the variability between replicate patches due to variability in the immediately surrounding habitats. Patch-orientation is potentially important for recruitment to artificial habitats, because animals dispersing via tidally-induced water currents move predominantly up and down shore in the direction of the current. It is thus likely that different orientations of experimental landscapes may collect different numbers of colonists [50]. To reduce the variability due to different patch-orientation, care was taken to attach all experimental landscapes so that the direction of prevalent swell/water-movement was approximately perpendicular to the row of units to be sampled in the experimental landscapes. We used three replicate landscapes of each treatment and replicated the entire design in another randomly chosen site 10′s m away.

Invertebrates rapidly colonize artificial turfs within 14 days of deployment [34], [51]. After 50 days, there are significant differences between assemblages colonizing habitats with different structural diversity [36]. Because of these previous findings, we sampled all experimental landscapes 50–55 days post-deployment, which was a reasonable compromise between period of colonization and durability of experimental landscapes on the shore. To prevent fauna and epiphytes being dislodged from a turf whilst it was being removed from the shore, we retrieved experimental landscapes using a grid of 5×5 cm^2^ plastic corers (similar to an ice cube tray), which isolated each individual piece of turf so that each one could be sampled separately, but simultaneously [36]. Each grid of corers was carefully placed over each side of the experimental landscapes and then pressed firmly down to enclose each of the turfs to be sampled. The screws were then undone, so that each of the eight pieces of turf was in a separate corer. Each corer was emptied into a separate plastic bag, guaranteeing that the epiphytes and fauna associated with each turf were completely recovered. All units were labelled and preserved in 7% formalin buffered in sea-water. Each turf was then washed in to a 500 µm sieve and all invertebrates sorted and counted under a binocular microscope at 16×magnification. All molluscs were identified to the finest possible taxonomic resolution, either species or morphospecies [52].

### Analysis of data

To test the prediction (hypothesis 1) that the diversity of benthic assemblages near the edge should differ from those in interiors of experimental landscapes, regardless of the composition of experimental landscapes, we analysed numbers of species in four different positions within experimental landscapes with different compositions. Composition was a fixed factor with four levels (i.e. Sparse, Dense, Sharp and Gradual); Site was a random factor with two levels; patches are the replicate experimental landscapes of each treatment and was a random factor nested in Composition and Site. Distance was fixed with four levels (i.e. 0–5, 5–10, 10–15 and 15–20 cm from the edge of a landscape. Two turfs from each distance from an edge were used as replicates for each experimental landscapes.

We compared numbers of species in particular types of turfs in landscapes of different composition. Significant differences between similar types of turfs in Sharp or Gradual landscapes would support the hypothesis (hypothesis 2) that assemblages differ depending on type of transitions. We tested this prediction separately for dense and for sparse turfs. In these analyses, the factor Composition had three levels (Sparse+Sharp and Gradual or Dense+Sharp and Gradual for each of the two types). Additionally, we tested the prediction (hypothesis 3) that the numbers of species in whole experimental landscapes would by affected by composition and types of edges. For this, we used the average of all eight sampled turfs from each of the 3 replicates of each experimental landscape. Composition was a fixed factor with four levels (Sparse, Dense, Sharp and Gradual); Site and Patch were as previously. For all analyses, when appropriate, data were transformed following homogeneity of variances test [Cochran's test; 53]. Student-Newman-Keuls (SNK) tests were used for *post*-*hoc* comparisons of the means. All univariate analyses tests were done using WinGMAV 5.0 (EICC, The University of Sydney).

We used multivariate analysis to test for differences among entire assemblages (as opposed to the number of species in the assemblages) to determine whether the assemblages colonizing different experimental landscapes depended on the types of transitions (hypothesis 4). Additionally, we tested whether assemblages colonizing particular types of habitats would be affected by the composition of the entire landscape (hypothesis 5). We tested these hypotheses (i.e. 4 and 5) using PERMANOVA [54] on Bray and Curtis (1957) multivariate distances on untransformed densities with Composition and Site as main factors, as described above. All multivariate procedures were done using PRIMER 6 with PERMANOVA+ (PRIMER-E, Plymouth, UK).

## Results

There were 4590 individuals of 55 species of molluscs in the experimental habitats; 13 of these species were singletons. About 51% of species were found across all distances from the edges of experimental habitats, whilst other species were less common or were rare and were found in different experimental habitats, without any apparent pattern. The most abundant species were the gastropods *Amphithalamus incidata* (Frauenfeld, 1867), juvenile *Austrocochlea porcata* (Adams, 1851), *Eatoniella atropurpurea* (Frauenfeld, 1867) and the bivalve *Lasaea australis* (Lamarck, 1818).

**Figure 2:**
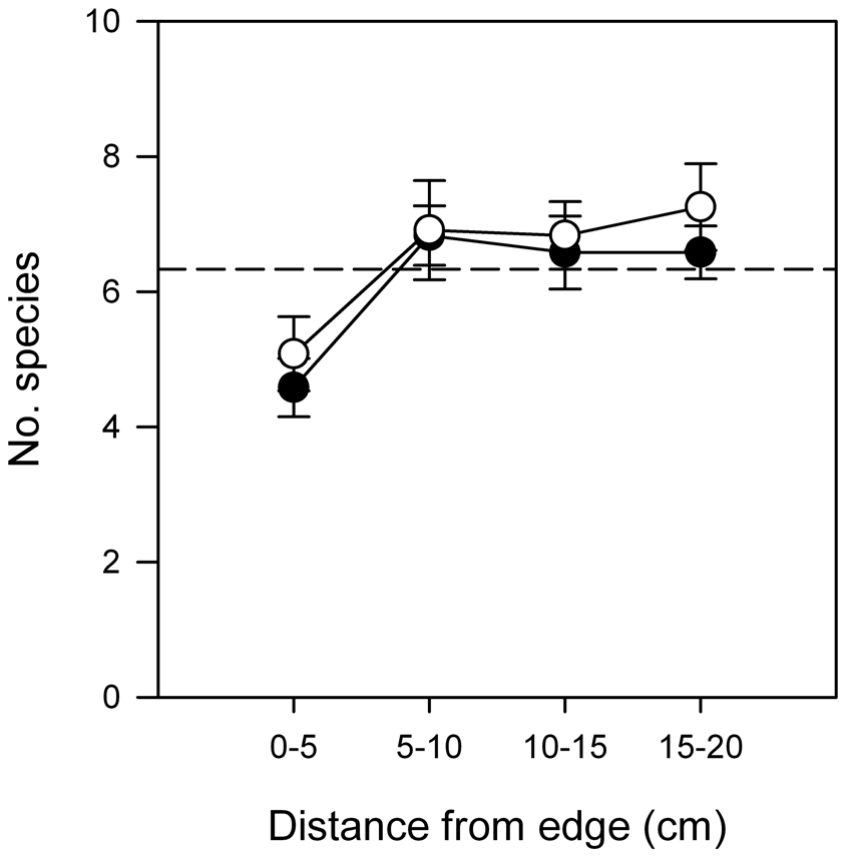
Effects of edges on numbers of species. Mean (±SE) numbers of species as a function of distance from edge of habitat (0–5, 5–10, 10–15 and 15–20 cm). For clarity in presenting the effect of distance from edge of habitat, the numbers of species were averaged across 2 habitat types (Sparse and Dense) and 3 replicate experimental landscapes in each site (1: •; 2: ○). Letters indicate means that are significantly different (SNK at *P* <0.05).

**Figure 3:**
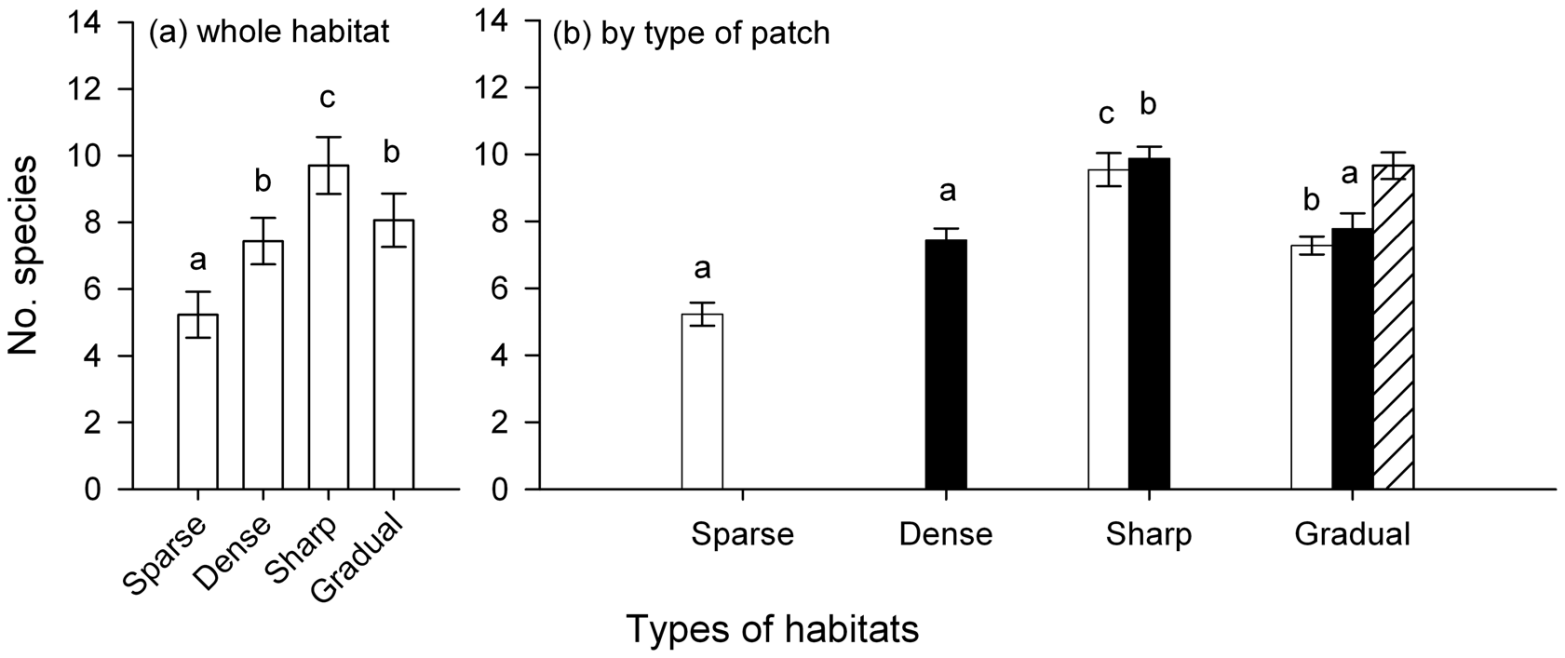
Effects of composition of experimental landscapes on numbers of species Mean (±SE) numbers of species in experimental landscapes with different composition (Sparse, Dense, Sharp and Gradual). Numbers of species are averaged across 2 sites and 3 replicate experimental landscapes. In (b), bars with different patterns indicate numbers of species in different types of turfs in landscapes of different composition: sparse (clear), dense (solid) and intermediate (striped) turfs. Letters indicate significant differences between means (SNK at *P*<0.05; analyses in Table 2.

Consistently fewer species were close to edges (0 – 5 cm) in any type of experimental landscape (SNK_LxD_ at *P* <0.05, Fig. 2; Table 1a), which supports hypothesis 1 that the effect of distance from edge and type would be independent of the type of landscape. There were more species in sparse turfs with sharp transitions than in similar turfs in Gradual or Sparse landscapes (Fig. 3b; Table 2c). Similarly, dense turfs with sharp boundaries also had more species, but there were no differences between Gradual and Sparse landscapes (SNK at *P* <0.05; Fig. 3b; Table 2c). Thus, the type of transition did influence the numbers of species in assemblages, but hypothesis 2 was only supported by numbers of species in sparse turfs.

**Table 1:** Analysis of numbers of species (means in Fig. 2) to test effects of distance from the edge and of composition of experimental landscapes.

Source	DF	MS	*F*	*P*
Composition = C	3	164.9	37.7	**<0.008**
Distance = D	3	46.0	10.8	**<0.05**
Site = S	1	0.3	0.1	>0.83
C×D	9	2.7	0.6	>0.76
C×S	3	4.4	0.8	>0.50
D×S	3	4.3	1.7	>0.18
C×D×S	9	4.4	1.7	>0.11
Patch (C×S)	16	5.4	2.4	**<0.006**
D×P(C×S)	48	2.5	1.1	>0.30
Residual	96	2.3		
Cochran's Test:		*C* = 0.14, *P*<0.05		
Transformation:		None		

Site is a random factor with two levels; Composition is a fixed factor with four levels (Sparse, Dense, Sharp or Gradual). Distance is fixed with four levels (0–5, 5–10, 10–15 and 15–20 cm from edge of experimental landscapes). Site is a random factor with 2 levels; Patch is a random factor nested in Composition and Site and has three levels; *n* = 2 turfs sampled at each distance in each treatment. Non-significant terms at *P >*0.25 that were not pooled, did not change the outcome of any tests relevant for the hypotheses being tested.

**Table 2:** Effects of composition of experimental landscapes on (a) number of species (i.e. across entire landscape), (b) average number of species in sparse turfs (Sparse, Sharp and Gradual), and (c) in dense turfs (Dense, Sharp and Gradual).

		(a) Entire landscapes			(b) Within Sparse turfs			(c) Within Dense turfs		
Source	DF	MS	*F*	*P*	MS	*F*	*P*	MS	*F*	*P*
Composition	3	20.61	37.65	**<0.01**	27.92	20.06†	**<0.001**	10.46	28.1†	**<0.001**
Site	1	0.03	0.05	>0.83	1.61	1.15†	>0.3	1.97	5.3†	**<0.04**
C×S	3	0.55	0.81	>0.51	0.79			0.42		
Residual	16	0.68			1.49			0.37		
Cochran's test:		*C* = 0.46, *P*>0.05			*C* = 0.42, *P*>0.05			*C* = 0.41, *P*>0.05		

† Tested against the pooled term: Residual+C×S.

Composition is a fixed factor with four levels in a) and three levels in b) and c). Site is random with two levels; *n*  = 3. All four variables are averages calculated for each experimental landscape. Means and SNK tests are in Fig. 2.

It is interesting to note that the average number of species in intermediate turfs in Gradual landscapes was greater than in dense or sparse turfs in the same habitats. When experimental landscapes were analysed as a whole, all of those with dense turfs – Dense, Sharp and Gradual – had more species than were in Sparse landscapes (SNK in Fig. 3a; Table 2a). Sharp landscapes had the largest average number of species, followed by Gradual and Dense landscapes, which supports hypothesis 3.

**Figure 4:**
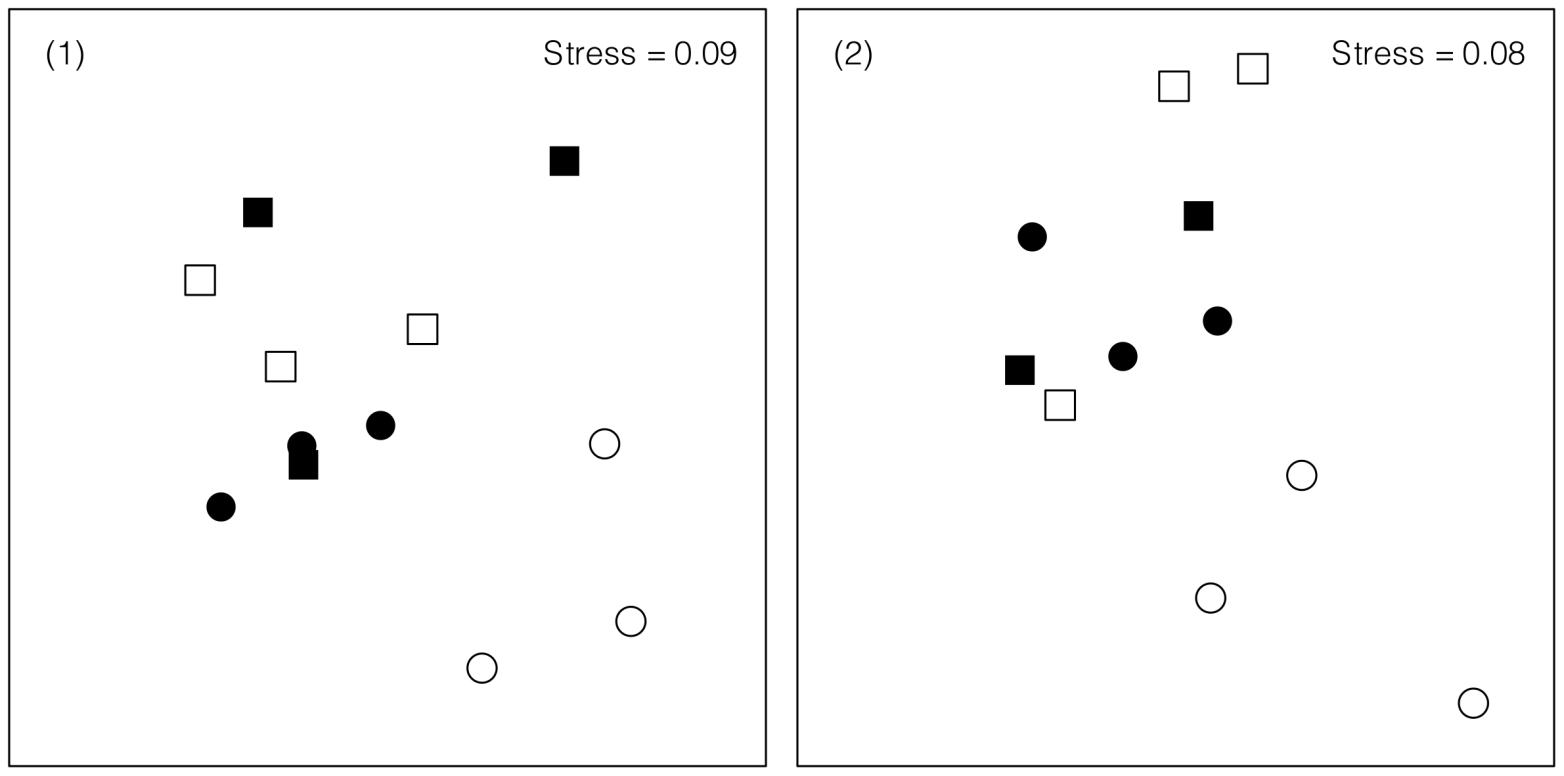
Effects of composition of experimental landscapes on benthic assemblages . nMDS ordination of Bray-Curtis distances of assemblages colonizing each experimental landscape with different composition using densities of species from *n* = 3 replicate patches in each site (1 or 2). Symbols indicate different types of landscapes: Sparse (□), Dense (○), Sharp (•) and Gradual (▪). Pair-wise tests of significance of differences between assemblages are in Table 3d.

Experimental landscapes with different composition were colonized by different assemblages (Fig. 4; PERMANOVA in Table 3), which supported hypothesis 4. When each type of turf was analysed separately (hypothesis 5), we found that assemblages in sparse or dense varied depending on the composition of the landscape in which they were included (Table 3). As would be expected given the difference in numbers of species, assemblages in Sparse landscapes were significantly different from those in Sharp, Gradual or Dense landscapes (pair-wise comparisons at *P*<0.05; Table 3a). Assemblages colonizing sparse turfs differed depending on the type of landscapes of which they were part (Table 3b). Sparse turfs in Sparse landscapes were significantly different from sparse turfs in Sharp or Gradual landscapes (pair-wise comparisons at *P*<0.05; Table 3b). In contrast, there were no differences among assemblages in dense turfs in the different landscapes (Table 3c).

**Table 3:** PERMANOVA examining Bray-Curtis distances between and within samples of assemblages in entire landscapes (a), sparse turfs (b) or dense turfs (c) in experimental landscapes and corresponding pair-wise comparisons of levels of factor Composition.

		PERMANOVA		Within and Between group dissimilarities					
		a) Entire habitats							
Source	DF	MS	*P*		Sparse	Sharp	Gradual		Dense
Composition = C	3	2323.50	**<0.001**	Sparse	50				
Site = S	1	871.72	>0.47	Sharp	55**	35			
C×S	3	889.09	>0.51	Gradual	**54** *	42	46		
Residual	16	950.28		Dense	**48** *	37	43		35
		b) Sparse turfs							
Source	DF	MS	*P*		Sparse	Sharp	Gradual		
Composition = C	1	1604.50	>0.21	Sparse	50				
Site = S	2	3156.40	**<0.002**	Sharp	65**	37			
C×S	2	725.47	>0.83	Gradual	**67** *	46	52		
Residual	12	1168.30							
		c) Dense turfs							
Source	DF	MS	*P*		Dense	Sharp	Gradual		
Composition = C	1	710.70	>0.72	Dense	35				
Site = S	2	1457.80	>0.20	Sharp	41	43			
C×S	2	571.18	>0.92	Gradual	46	47	50		
Residual	12	1084.80							

* indicates *P*<0.05 and ** indicates *P*<0.005

Site is random with two levels; Composition is a fixed factor four levels in a); three levels in b) and c); *n* = 3. Entries are the mean distances between each pair in each test of Composition, with bold entries with asterisks being significant at *P* = 0.05. Multivariate patterns for entire habitats are shown in Fig. 4.

## Discussion

Our main findings were that: (1) fewer species occurred close to edges (0–5 cm) of experimental landscape; (2) differences in composition of habitat had major effects on assemblages in different types of habitats, although (3) reductions in numbers of species closer to edges were independent of the composition of landscape; (4) sharp as opposed to gradual transitions influenced the magnitude of interactions among different types of habitats and finally (5) intermediate turfs (i.e. with intermediate structure) had more species than did sparse or denser turfs.

### “Living on the edge”

Our results showed that edge-effects were independent of the composition of experimental landscapes because there were consistently fewer species closer to edges (0–5 cm) in all types of experimental landscapes. Taking into account that there were differences in numbers of species between different types of landscape, the proportional magnitude of edge-effects was consistent. Only 15 to 25% of the species inside experimental landscapes were found near the edges. This might be explained by greater accumulations of individuals in the middle of experimental landscapes (i.e. an “interior effect”), as opposed to an edge-effect, that is general to many types of experimental landscapes. Larvae which disperse passively through the water-column [e.g. most marine gastropods, 55] are more likely to settle in the middle than on the edges of experimental landscapes simply due to hydrodynamic patterns [56]. Edges and interiors of habitats have different current-speeds or turbulences [57].

Alternatively, there may be differences in boundary layers [56], which have been offered as explanations for differences between edges and interiors of other artificial surfaces [20] or cleared areas [58]. Such differential settlement between edges and interiors of patches has been proposed to explain differences in assemblages in different-sized patches [58], [59], [60]. In seagrass beds, however, size, shape and orientation of patches can determine the post-settlement distributions, even though there is increased recruitment near edges as a result of reductions in water-flow at edges [e.g. 50,61,62]. Although such information is not available for the turf-like habitats used here, it is likely that some of these processes are also occurring at smaller scales. This could explain the differences in numbers of species between edges and interiors of experimental landscapes. There is, however, a lack of consistency in edge-effects in some other marine habitats, such as seagrass beds. Some are neutral, i.e. have no effect [or neutral edge effects, 25,e.g. positive, 26,negative, 28] in terms of numbers of species or numbers of individuals of some species. Here, there was a consistent decrease in numbers of species at the edges of all the different types of landscapes. It is clear that comparable experiments will be required in other habitats before any general understanding can be gained.

### “Living in a transition habitat”

Independently of the effects of edges, the composition of experimental landscapes had major effects on the structure of benthic assemblages. Denser turfs were colonised by greater numbers of species, which probably explains why all landscapes with dense turfs (i.e. Dense, Sharp and Gradual) had more species than did Sparse landscapes. Sharp landscapes had the largest average number of species, followed by Gradual and Dense landscapes. This result was expected because landscapes with different types of transitions between habitats (Sharp and Gradual) are likely to offer different types of resources, therefore sustaining more species [e.g. 36,63,64,65].

Denser turfs had greater density and surface area of fronds and were expected to have more individuals [66]. Interestingly, sparse turfs clearly had fewer species than did denser turfs in monotypic areas, although there were no differences in numbers of species between these two types of turfs in the heterogeneous landscapes (i.e. Sharp and Gradual). This is a clear indication that assemblages colonizing experimental landscapes made of different types of habitats interacted independently of the type of border. In addition, there were more species in sparse turfs in heterogeneous landscapes (i.e. Dense or Gradual) than in monotypic landscapes. This shows that there were interactions between assemblages colonizing the two sides of an edge, increasing the diversity of assemblages which colonized those landscapes that otherwise would have been expected to maintain fewer species. These results re-emphasise the importance of particular types of habitats as opposed to numbers of types of habitats (or “habitat diversity”), to support diverse assemblages in heterogeneous landscapes [36], [67].

At the scale of the whole experimental landscape, there were more species in landscapes with sharp transitions (i.e. Sharp) than in landscapes where transitions are gradual. It is interesting to note that there were more species in intermediate turfs in Gradual landscapes than in dense or sparse turfs. Intermediate turfs have some structural properties in common with other types of turfs and may therefore provide the habitat for species occurring in either of the other two (dense or sparse). Numbers of species in intermediate turfs should, however, have been “intermediate” between those in the other two types of turfs [35]. Possibly, intermediate turfs are different habitats on their own right (i.e. have different structural complexity and resources). Intermediate turfs may have be an ecotone [sensu 10] with a unique set of characteristics that are a function of interactions between adjacent habitats. Such ecotones often sustain greater diversity of species than found in the assemblages in adjacent habitats [see reviews by 7,9,11]. Typical examples are edges of forests, which have distinctive micro-climates and are generally rich in micro-habitats [4]. In our experiment, intermediate turfs may be colonized by greater numbers of species as a result of their “intermediate” position in the experimental landscapes or due to the “intermediate” structure that such a component of a landscape offers. In fact, it has been shown that the relationship between structural complexity and diversity of benthic macro-invertebrates in coralline turfs is not necessarily linear [41], which could explain why an intermediate habitat type would have greater numbers of species. Future studies should address this issue by including experimental landscapes to disentangle the mechanisms underlying the role of transitions in the heterogeneous habitats.

We experimentally manipulated composition (i.e. number and types of habitats) together with types of transitions within experimental landscapes. The responses of organisms that are<3 mm in length are relevant to studies at larger scales and in different systems. Yet, studying species' responses at the appropriate biological and ecological scales will make important contributions to discussions of the role of heterogeneity and ecological boundaries in natural habitats [e.g. 48,see review on the value of microcosms, 68]. While classical examples of major ecological boundaries such as the vast transition zones (or ecotones) between African rainforest and savanna, often more than hundreds of km wide [69], are likely to share some general characteristics with other ecological boundaries, their specificities make it impossible to establish clearly valid comparisons with any other habitat. Better understanding of ecological boundaries can only arise from comparisons across many types of systems and their associated assemblages of species. This understanding has been, however, marred by a lack of consistency between edge- or boundary-related effects [see review by 3].

Our study contributes to understanding of the role of ecological boundaries in natural landscapes using an experimental approach that can potentially aid in the interpretation of observational studies where the experiments may be impossible (e.g. because of intractable spatial scale of habitats). Our artificial "landscapes" allowed us to explore different aspects of heterogeneous habitats by explicitly defining what is "patch" or "landscape" according to the scale at which these organisms are known to respond.

Our results are therefore relevant to any conceptual framework where the scale of the study is defined and is relevant to the system being studied [see review in 9,70]. A major conclusion from our study is that it is important to understand conceptually the roles of patches versus the whole landscape and the boundaries b etween the patches and those of the landscape itself Our results are therefore relevant to any conceptual framework where the scale of the study is defined and is relevant to the system being studied [see review in 9,70]. A major conclusion from our study is that it is important to understand conceptually the roles of patches versus the whole landscape and the boundaries between the patches and those of the landscape itself. Clearly, these can only be understood if they are carefully defined in relation to the organisms and habitats being studied. These considerations should be incorporated into future experimental studies about the roles of ecological boundaries in natural habitats [e.g. 48,see review on the value of microcosms, 68]. Clearly, these can only be understood if they are carefully defined in relation to the organisms and habitats being studied. These considerations should be incorporated into future experimental studies about the roles of ecological boundaries in natural habitats.
