# Correction: Macrofaunal Responses to Edges Are Independent of Habitat-Heterogeneity in Experimental Landscapes

**DOI:** 10.1371/annotation/c6bcddf5-835c-417f-a32e-201537ad4924

**Published:** 2013-04-09

**Authors:** Miguel G. Matias, Ross A. Coleman, Dieter F. Hochuli, Antony J. Underwood

There was an error in the funding statement. The correct funding information is:

This work was supported by funds from the Fundação para a Ciência e Tecnologia (FCT) SFRH/BD/27506/2006 to MGM, by grants from the Australian Research Council to AJU and additional funds from the EICC. The funders had no role in study design, data collection and analysis, decision to publish, or preparation of the manuscript. 

